# Patient-reported outcomes in adults with type 3c diabetes: a scoping review across aetiologies and pancreatic resection extent

**DOI:** 10.3389/fendo.2026.1963887

**Published:** 2026-09-14

**Authors:** Ana Meden, Nika Aleksandra Kravos Tramšek

**Affiliations:** Department of Endocrinology and Diabetology, University Medical Centre Maribor, Maribor, Slovenia

**Keywords:** continuous glucose monitoring, pancreatectomy, pancreatogenic diabetes, patient-reported outcomes, quality of life, sarcopenia, scoping review, type 3c diabetes

## Abstract

**Background:**

Type 3c diabetes is a heterogeneous secondary diabetes caused by pancreatic disease, injury or resection. Glycaemic outcomes alone do not capture the combined effects of insulin deficiency, impaired counter-regulation, exocrine insufficiency, pain, altered nutrition and treatment burden. We mapped standardized patient-reported outcome measures (PROMs) across aetiologies and pancreatic resection extent.

**Methods:**

MEDLINE via PubMed, Scopus and Web of Science Core Collection were searched from inception to 23–24 July 2026, supplemented by public trial registries and targeted citation checking. A scoping review framed by Population–Concept–Context followed JBI guidance and PRISMA-ScR. An automation-assisted title/abstract pathway was followed by independent report-level adjudication by two reviewers.

**Results:**

The searches yielded 5,132 records and 3,372 unique database records after deduplication. Fifty-six reports underwent independent report-level adjudication; one was not retrieved, 15 were excluded and 40 were included. In a reproducible *post hoc* random validation sample of 200 of 3,321 early exclusions, exact and binary reviewer agreement were 100% (Cohen’s kappa = 1.00); three records marked unclear by both reviewers were excluded after targeted adjudication, leaving no eligible record in the sample (0/200; Wilson 95% confidence interval 0–1.88%). Twenty-six included reports concerned pancreatic resection or an insulin-treated TPIAT subgroup, 11 cystic-fibrosis-related diabetes, two diabetes secondary to chronic pancreatitis and one fibrocalculous pancreatic diabetes. Generic health and physical function were often impaired after total pancreatectomy, whereas some comparator studies reported similar PAID, ADDQoL or treatment-satisfaction results to type 1 or other insulin-treated diabetes. No report used SARC-F. Only six reports were available to the authors in full text; the favorable technology signals depended on abstract-based reports and were therefore hypothesis-generating.

**Conclusions:**

Patient-reported burden in type 3c diabetes is multidimensional and measurement remains fragmented. Future studies should combine complementary measures and report aetiology, diabetes timing and total versus partial resection explicitly.

## 1 Introduction

Type 3c diabetes mellitus is secondary to pancreatic disease, injury or resection. The related term diabetes of the exocrine pancreas is used in classification and epidemiological literature, although terminology and diagnostic boundaries differ across settings. Chronic or recurrent pancreatitis, pancreatic neoplasia, partial or total pancreatectomy, pancreatic trauma, cystic fibrosis, haemochromatosis, fibrocalculous pancreatic diabetes and congenital pancreatic disorders may lead to diabetes through partly distinct mechanisms (1–4). Misclassification as type 1 or type 2 diabetes makes both epidemiology and patient-experienced burden difficult to define (2, 3).

The phenotype extends beyond insulin deficiency. Impaired pancreatic glucagon responses, altered nutrient digestion and absorption, exocrine insufficiency, pain, gastrointestinal reconstruction, cancer-related symptoms and nutritional depletion may coexist with glucose variability and hypoglycaemia (1, 4, 5). Fear of hypoglycaemia may restrict physical activity, while pain, malabsorption and weight loss may reduce muscle mass and functional reserve. HbA1c and continuous glucose monitoring metrics therefore cannot represent the full patient burden.

Resection extent is a plausible source of heterogeneity. Total pancreatectomy produces an apancreatic state with complete loss of pancreatic insulin, glucagon and pancreatic polypeptide secretion, whereas partial resection preserves a variable amount of endocrine and exocrine tissue (5). Type 1 diabetes is a useful comparator for severe insulin deficiency but retains pancreatic anatomy; type 2 diabetes is dominated by insulin resistance with variable beta-cell failure. Neither comparator reproduces the combined consequences of pancreatic disease, surgery and exocrine insufficiency.

PROMs provide direct information about symptoms, functioning, health status and quality of life (6). EQ-5D-5L supports generic health-status and utility comparisons (7), ADDQoL captures the individualized effect of diabetes on valued life domains (8), and SARC-F is a brief patient-reported screen for sarcopenia risk and adverse functional outcomes rather than a diagnostic test (9). Existing reviews have focused largely on total pancreatectomy or surgical populations (10, 11).

The primary objective was to map standardized PROM domains and instruments used in adults with type 3c diabetes across aetiologies and treatment modalities. Secondary objectives were to compare total and partial resection, summarize direct comparisons with type 1 or type 2 diabetes, examine patient-reported effects of glucose technologies, evaluate the use of EQ-5D-5L, ADDQoL and SARC-F, and identify priorities for a patient-centered outcome set.

## 2 Methods

### 2.1 Design and reporting

A scoping-review design was selected because the anticipated evidence was heterogeneous in aetiology, study design, intervention and measurement instrument. The approach followed JBI guidance (12) and PRISMA-ScR (13). No meta-analysis was planned. The protocol was not prospectively registered; an internal eligibility codebook and charting form were developed before final report-level adjudication.

### 2.2 Eligibility criteria

Studies were eligible when they reported quantitative primary research in adults aged at least 18 years with clearly defined type 3c diabetes caused by any pancreatic disease or intervention; used at least one named standardized patient-completed PROM; and provided separately extractable findings for the eligible group. Randomized, non-randomized, cohort, case-control, cross-sectional and mixed-methods studies were eligible when the quantitative PROM component was separable. Reviews, editorials, protocols, single case reports, qualitative-only studies, insufficient conference abstracts and studies using only unnamed or custom questionnaires were excluded.

The review question was framed using Population–Concept–Context (PCC): adults with type 3c diabetes (population); standardized patient-reported outcome instruments and domains (concept); and all pancreatic aetiologies, resection extents and diabetes-management settings (context). Quantitative primary studies were the eligible evidence sources.

Type 3c diabetes was operationalized according to aetiology and timing. Total pancreatectomy was treated as etiologically certain postoperative apancreatic diabetes. After partial resection, eligibility required postoperative or new-onset diabetes, or diabetes explicitly attributed to pancreatic disease, with separately extractable PROM findings. Chronic- or recurrent-pancreatitis reports required diabetes described as pancreatogenic, post-pancreatitis or secondary to pancreatitis; concurrent diabetes without temporal or etiological attribution was excluded. Cystic-fibrosis-related and fibrocalculous pancreatic diabetes were accepted as established aetiological categories when adult results were separable. TPIAT reports were eligible only for a separately reported diabetic or insulin-treated subgroup. Pancreatic cancer, pancreatitis or resection alone was insufficient without the relevant diabetes definition, except after total pancreatectomy. Mixed paediatric–adult cohorts required adult-only findings.

Operational eligibility criteria are summarized in **Table 1**.

**Table 1:** Operational PCC eligibility criteria.

PCC domain	Included	Excluded or restricted
Population	Adults aged at least 18 years with clearly defined type 3c diabetes of any pancreatic aetiology.	Type 1, type 2, gestational or undefined diabetes; mixed ages without adult-only data.
Concept	At least one named standardized patient-completed PROM, with quantitative results separable for the eligible group.	Only clinical/CGM outcomes, unnamed or custom questionnaire, qualitative-only data or vague quality-of-life statement.
Context	All pancreatic aetiologies and management settings; total resection; partial resection with attributable postoperative diabetes; TPIAT with a separable diabetic/insulin-treated subgroup.	Pancreatic disease or surgery without attributable diabetes; mixed surgical/TPIAT cohort without separable diabetes status.
Evidence source	Randomized, non-randomized, cohort, case-control, cross-sectional or mixed-methods primary study with separable quantitative PROM data.	Review, editorial, protocol, single case report or insufficient conference abstract.

### 2.3 Information sources and search strategy

MEDLINE via PubMed, Scopus and Web of Science Core Collection were searched from inception on 23–24 July 2026 without date or language limits. ClinicalTrials.gov and the WHO International Clinical Trials Registry Platform were searched on 24 July 2026 to identify ongoing or unpublished work. Embase, CINAHL and APA PsycINFO were unavailable because institutional access was not available. Searches combined type 3c diabetes and aetiology terms with quality-of-life, PROM, questionnaire, treatment-burden, hypoglycaemia-awareness and named-instrument terms. Davidsen et al. (14) was used as a sentinel record. Complete strategies are provided in **Supplementary Appendix 1**.

Search yields and source status are summarized in **Table 2**.

**Table 2:** Electronic searches.

Source	Date	Records	Status
MEDLINE via PubMed	24 Jul 2026	1,376	Completed
Scopus	23 Jul 2026	2,621	Completed
Web of Science Core Collection	24 Jul 2026	1,135	Completed
ClinicalTrials.gov	24 Jul 2026	202	Supplementary registry search
WHO ICTRP	24 Jul 2026	25 trial groups	Supplementary registry search
Embase, CINAHL, APA PsycINFO	—	—	Unavailable; reported as limitation

### 2.4 Record management and study selection

Bibliographic exports were normalized and deduplicated using PMID, DOI and conservative exact-title rules. Fuzzy-title pairs were inspected and not merged automatically. An automation-assisted workflow using rule-based eligibility signals and a language model ranked records for detailed title/abstract inspection. The initial workflow identified 128 higher-priority or uncertain records; a subsequent quality audit reinstated 17 database records and located five reports outside the database exports. The combined automation-assisted pathway marked 3,321 database records ineligible and retained 51 database reports for report-level review.

Following peer-review concern about early exclusions, a *post hoc* validation sample of 200 records was drawn reproducibly from the 3,321-record automation-assisted exclusion pool using the deterministic seed “Type3c_PROMs_posthoc_validation_2026-08-27_v1”. Ana Meden and Nika Aleksandra Kravos Tramšek independently screened titles and abstracts with the prespecified criteria and without access to each other’s decisions. Decisions were recorded as exclude, retain or unclear; retain/unclear records advanced to targeted public-source or full-text adjudication. Exact three-category agreement, binary agreement and Cohen’s kappa were calculated. Because the sample contained no known eligible records, this exercise estimated the potentially missed-eligible proportion rather than screening sensitivity.

Ana Meden and Nika Aleksandra Kravos Tramšek independently assessed 56 reports (51 from databases and five from supplementary searching) using the same operational criteria. Decisions were compared after independent completion. All decisions were concordant. One report could not be retrieved; eligibility could not be confirmed because the named standardized PROM was not available in public metadata. A single primary exclusion reason was recorded for each excluded report.

### 2.5 Data charting and synthesis

A standardized form captured citation, design, aetiology, pancreatic procedure, operational diabetes definition and timing, relevant sample, comparator, intervention, follow-up, PROM name and domain, principal finding, evidence basis, cohort overlap and eligibility restriction. Restricted inclusion was used when only a defined diabetes subgroup or instrument was interpretable. Full-text availability was recorded explicitly; unavailable variables were coded as not reported and were not inferred. PROMs were grouped as generic HRQoL; diabetes-specific quality of life; diabetes distress; treatment satisfaction; hypoglycaemia awareness or fear; pancreas-, cancer- or cystic-fibrosis-specific HRQoL; emotional wellbeing; symptoms; and physical or social functioning.

Findings were synthesized descriptively by aetiology, resection extent, PROM domain and study purpose. Heterogeneous instruments, populations and reporting formats precluded meaningful pooling; SWiM principles informed the narrative structure (15). Because the purpose was evidence mapping rather than effect estimation, no formal design-specific risk-of-bias score was assigned. Instead, the master evidence map records report-level methodological limitations, including sample size, design, response, cohort overlap, diabetes definition, evidence accessibility and separation of the relevant type 3c subgroup.

### 2.6 Use of artificial intelligence

OpenAI ChatGPT/Codex (GPT-5, OpenAI; accessed July–August 2026) was used for language editing, document-formatting assistance, query translation, and preliminary organization, prioritization and consistency checking of search records. It did not independently make final report-level eligibility, data-extraction or interpretation decisions. Both authors independently reviewed and confirmed eligibility decisions for all 56 reports subjected to report-level assessment, including all 40 included reports, and independently completed the 200-record *post hoc* validation sample. The authors reviewed and verified the entire manuscript and take full responsibility for its content.

## 3 Results

### 3.1 Study selection

The three bibliographic searches identified 5,132 records: 1,376 from PubMed, 2,621 from Scopus and 1,135 from Web of Science. Exact deduplication removed 1,760 rows, leaving 3,372 unique database records. The automation-assisted screening pathway retained 51 database reports for independent report-level assessment; five further reports were added through the targeted audit and citation searching. One of 56 reports was not retrieved. Of 55 assessed reports, 40 met the eligibility criteria and 15 were excluded. The full report-level decisions are supplied in **Supplementary Tables S1**, **S2**. The study-selection process is shown in **Figure 1**.

**Figure 1:**
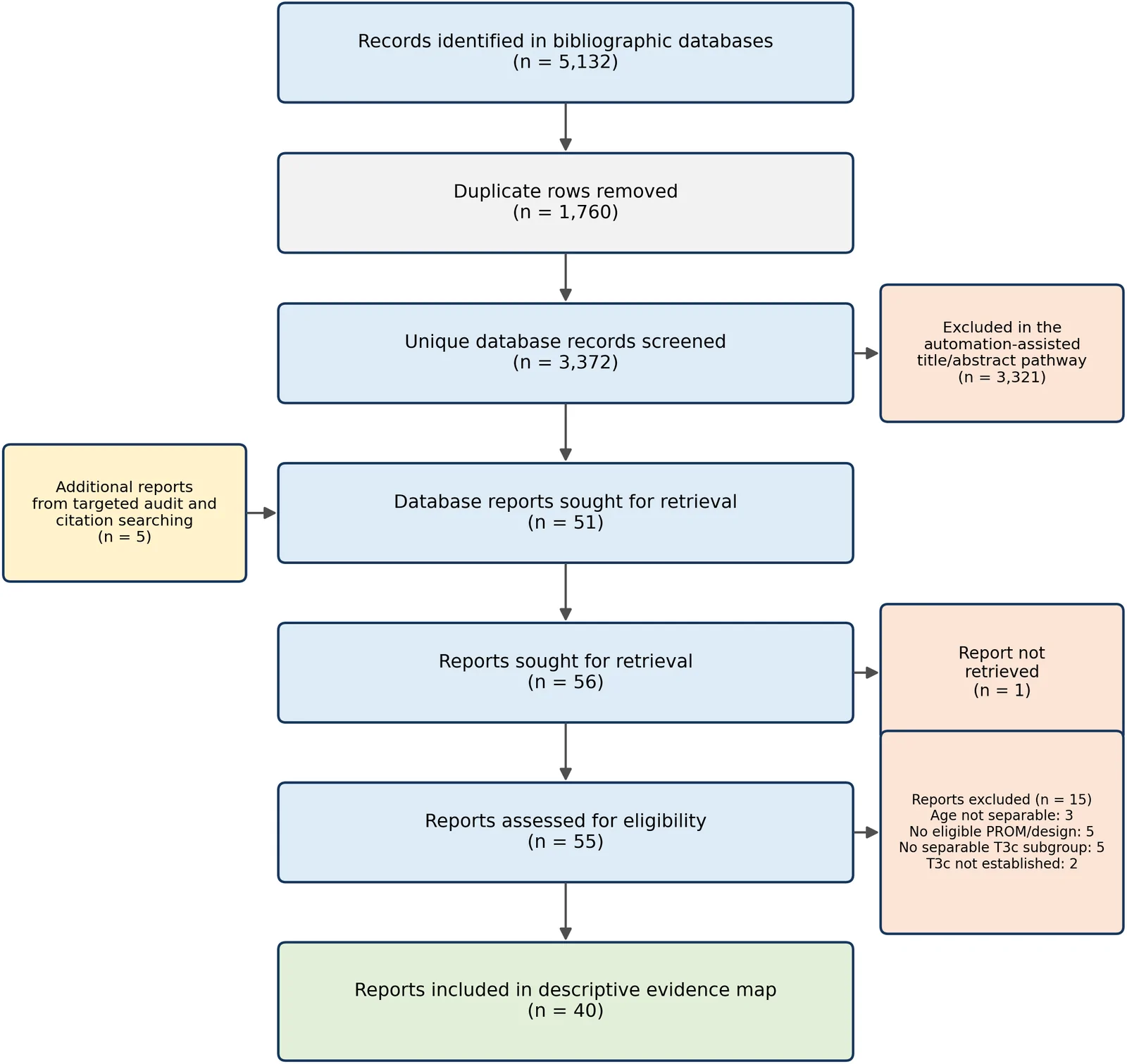
PRISMA-ScR flow diagram of study selection. The searches identified 5,132 database records; after removal of 1,760 duplicates, 3,372 unique records were screened. Fifty-six reports were sought, one was not retrieved, 55 were assessed for eligibility and 40 were included in the descriptive evidence map. PRISMA-ScR, Preferred Reporting Items for Systematic Reviews and Meta-Analyses extension for Scoping Reviews.

In the *post hoc* validation sample, both reviewers independently classified 197 records as exclude and the same three as unclear; neither retained a record at title/abstract stage. Exact three-category and binary agreement were 100% (Cohen’s kappa = 1.00). Targeted adjudication excluded all three unclear records because of an absent standardized PROM, an inadequately attributed diabetes aetiology, or non-separable PROM findings. Thus, no eligible record was identified in the sample (0/200; Wilson 95% confidence interval 0–1.88%). This result does not directly estimate screening sensitivity.

### 3.2 Characteristics of the evidence map

The 40 included reports were published from 2005 to 2026. Twenty-six reports concerned pancreatic resection or a separately interpretable insulin-treated TPIAT subgroup, 11 concerned CFRD, two concerned diabetes secondary to chronic pancreatitis and one concerned fibrocalculous pancreatic diabetes. The evidence included one randomized crossover trial, small prospective technology and education studies, and predominantly cross-sectional or retrospective surgical cohorts (14, 16–54). Several reports probably represented overlapping surgical cohorts; findings were therefore mapped at report level and participants were not summed.

Seven reports directly compared total with partial or subtotal resection (20, 24, 25, 31, 38, 43, 49). Five included type 1 or other insulin-dependent diabetes comparators (26, 29, 30, 33, 34); no report directly compared confirmed type 3c with type 2 diabetes. Three studies evaluated CGM or automated insulin delivery as interventions (14, 16, 17), and one evaluated structured education (18).

### 3.3 PROM instruments and domains

Instrument use was heterogeneous. In the adjudicated evidence map, EORTC QLQ-C30 was recorded in 18 reports and QLQ-PAN26 in 11, predominantly after pancreatic surgery. ADDQoL appeared in six reports, PAID/PAID-20 in seven, DTSQ in three, EQ-5D-5L in four and CFQ-R in four. Additional instruments included SF-36/SF-12, PANQOLI, CFAbd-Score, HADS, GAD-7, PHQ-8/PHQ-9, Fatigue Symptom Inventory, Diabetes Self-Management Questionnaire, Clarke hypoglycaemia-awareness questionnaire, PGIC, physical-activity recall and self-care measures (14, 16–34, 37–54). No study validated a PROM specifically for type 3c diabetes.

### 3.4 Outcomes after pancreatic resection

Total-pancreatectomy reports generally identified residual impairment in generic, physical or pancreas-specific health compared with population norms (23, 26, 27, 29, 34–39, 50, 51, 53, 54). In PANORAMA, global health was 73 versus 78 and the EQ-5D index 0.83 versus 0.87 in the general population (26). Stoop et al. reported EORTC global quality of life of 66.7 versus 76.4, although scores were similar to the patients’ preoperative state (23). Nutritional status, exocrine replacement, symptoms and follow-up time varied substantially across reports.

Diabetes-specific burden was less uniformly impaired. In the direct comparator reports, PAID diabetes-distress scores, ADDQoL average weighted impact or individual life-domain effects, and DTSQ treatment-satisfaction scores were often similar to type 1 or other insulin-treated diabetes (26, 29, 30, 34). By contrast, generic physical and general health, pancreas-specific symptoms and selected dietary or activity domains were not consistently similar. These comparisons do not isolate diabetes from surgery, cancer, pain or exocrine insufficiency.

### 3.5 Total versus partial resection

Comparative findings were domain-specific. Some studies reported similar global quality of life after total and partial resection, while total pancreatectomy was associated with worse diabetes-specific quality of life, physical function, fatigue interference, insomnia, dietary restriction or confidence about the future (20, 24, 25, 31, 38, 43, 49). Restricted inclusion was necessary when partial-resection findings combined patients with and without postoperative diabetes (21, 22, 43, 47). The available reports therefore do not support a simple conclusion that every PROM domain is worse after total pancreatectomy.

### 3.6 Chronic pancreatitis and fibrocalculous pancreatic diabetes

In a randomized crossover trial of 30 participants with chronic pancreatitis and insulin-treated diabetes, CGM improved several glycaemic metrics, but EORTC QLQ-C30, PGIC and Clarke questionnaire results did not differ significantly between treatment periods; 25 participants supplied PROM data (14). A specialist-clinic cohort with diabetes secondary to chronic pancreatitis identified substantial pancreas-specific and psychological burden, although the psychological PROM subset was small (19).

The single fibrocalculous pancreatic diabetes report included 60 adults and used the standardized eight-item Clarke questionnaire together with CGM-derived measures. Forty-four participants were classified as having impaired hypoglycaemia awareness, identifying a clinically important patient-reported safety concern in this aetiology (46).

### 3.7 Cystic-fibrosis-related diabetes

Two small uncontrolled CFRD technology reports described improvements in diabetes-specific PROMs alongside glycaemic outcomes: DTSQ increased and ADDQoL became less negative after automated insulin delivery (16), while PAID decreased during CGM use (17). These reports were available to the authors as structured abstracts rather than full texts, so the numerical signals are treated as hypothesis-generating.

The wider CFRD literature identified treatment burden, gastrointestinal symptoms, psychosocial burden, self-management barriers, physical activity and sensory symptoms using CFQ-R, CFAbd-Score, PAID, HADS, self-management and symptom questionnaires (18, 28, 32, 40, 42, 44, 45, 48). In the direct type 1 diabetes comparison, adults with CFRD described a less negative diabetes-related quality-of-life impact and fewer severe neuroglycopenic experiences, although both groups experienced hypoglycaemia (33).

### 3.8 EQ-5D-5L, ADDQoL and SARC-F

EQ-5D-5L remained uncommon and was concentrated in surgical comparisons (22, 26, 38, 49). ADDQoL was the most widely distributed diabetes-specific quality-of-life instrument across CFRD, total pancreatectomy and resection comparisons (16, 21, 27, 29, 31, 34). It detected negative effects of diabetes on selected life domains; in some direct comparator studies, ADDQoL average weighted impact or domain patterns were similar to other insulin-treated diabetes, whereas generic physical health and pancreas-specific symptoms were not consistently similar.

No report used SARC-F. Physical activity, fatigue, nutrition, dietary restriction and physical functioning appeared in several instruments (25, 27, 37, 40, 48), but these constructs are not interchangeable with a validated sarcopenia screen. The absence of SARC-F is therefore a measurement gap, not evidence that sarcopenia-related burden is absent.

### 3.9 Critical-appraisal considerations

No study-level summary risk-of-bias rating was assigned. The expanded master evidence map instead records design, sample, comparator, follow-up, diabetes definition, evidence basis and key limitations for each report. Recurrent concerns included small samples, cross-sectional or retrospective designs, low or incompletely reported response, overlapping surgical cohorts, survivorship selection, heterogeneous diabetes definitions, incomplete separation of type 3c subgroups and limited full-text accessibility. These features precluded causal comparison and meta-analysis.

### 3.10 Evidence accessibility and sensitivity analysis

Six of the 40 included reports were evaluated from accessible full text; for 34, eligibility and at least part of the descriptive charting relied on structured abstracts, public metadata or secondary bibliographic evidence. A sensitivity analysis restricted to the six full-text reports retained representation of the four principal aetiological settings and supported the high-level conclusions that instrument selection was heterogeneous, resection-specific reporting was necessary and SARC-F had not been used. It did not retain the favorable CGM or automated-delivery signals from the abstract-based CFRD reports; those signals are therefore not treated as established effects.

## 4 Discussion

### 4.1 Principal findings

Patient-reported research in type 3c diabetes is fragmented, instrument-rich but validation-poor, and dominated by selected surgical survivors. The most consistent pattern was a separation between overall health and diabetes-specific burden. Total-pancreatectomy cohorts often reported reduced general or physical health, yet some comparator reports found similar PAID diabetes distress, ADDQoL impact or DTSQ treatment satisfaction to type 1 or other insulin-treated diabetes. These signals are not universal and should not be generalized across instruments or aetiologies. A single diabetes-specific score cannot represent the combined effects of pancreatic disease, exocrine insufficiency, pain, nutritional compromise and surgery.

### 4.2 Resection extent and comparators

Direct comparisons did not show uniformly poorer PROMs after total pancreatectomy. Differences emerged in diabetes-specific quality of life, fatigue, physical function, sleep, dietary restriction and future confidence, while global quality of life was often similar. Interpretation is limited by operative indication, tumor burden, pre-existing diabetes, residual tissue quality, exocrine insufficiency, altered anatomy and follow-up. Future reports should distinguish total, completion-total and specific partial resections and report residual endocrine function rather than use procedure name alone as a mechanistic proxy.

Type 1 diabetes is useful for comparison of severe insulin deficiency and intensive treatment but does not reproduce exocrine insufficiency, reconstruction, cancer or chronic pancreatic pain. No direct PROM comparison with confirmed type 2 diabetes was identified, despite the clinical problem of misclassification.

### 4.3 Measurement implications

No single instrument covered all relevant constructs. EQ-5D-5L enables population and health-utility comparison but may be insensitive to diabetes-management change. ADDQoL, PAID and DTSQ capture diabetes-specific impact, distress and satisfaction but may miss exocrine symptoms, pain, altered eating and physical decline. Pancreas- and CF-specific instruments capture underlying-disease burden but may not isolate diabetes. A complementary battery is therefore preferable.

The evidence supports combining a generic health-status measure with a diabetes-specific measure and a separate assessment of physical or functional status. EQ-5D-5L and ADDQoL are plausible complementary instruments. SARC-F may be a pragmatic candidate for prospective evaluation, but it has not been used or validated in type 3c diabetes and cannot be recommended as an established component of a core outcome set. If used, it should be paired with objective muscle strength, performance or body-composition measures.

### 4.4 Technology and patient-centred care

The recent CFRD reports raise the hypothesis that diabetes-specific PROMs may improve with CGM or automated insulin delivery, whereas the randomized chronic-pancreatitis trial improved glycaemic metrics without a detectable generic PROM change. Because the favorable CFRD reports were small, uncontrolled and not available to the authors in full text, efficacy cannot be inferred. Future technology trials should prespecify treatment satisfaction, distress and hypoglycaemia confidence in addition to generic HRQoL and test whether benefit differs after total and partial resection.

Patient-centred care also extends beyond glucose-monitoring technology. Insulin regimen, pancreatic enzyme replacement, nutrition, pain treatment and the choice of glucose-lowering therapy may influence symptoms, eating, treatment satisfaction and quality of life. Evidence for non-insulin agents in type 3c diabetes remains limited and treatment must account for residual endocrine function, nutritional status and pancreatic disease. In people with obesity or type 2 diabetes, a large observational analysis associated GLP-1 receptor-agonist initiation with lower all-cause mortality but a small early increase in acute pancreatitis risk, underscoring individualized risk–benefit assessment in patients with pancreatic vulnerability (55); these findings cannot be directly extrapolated to type 3c diabetes.

### 4.5 Strengths and limitations

Strengths include a cross-aetiology question, explicit separation of total and partial resection, a stringent named-PROM criterion, searches in three major bibliographic databases and two public registries, conservative deduplication, and fully concordant independent report-level decisions by two reviewers. The synthesis keeps generic, disease-specific and diabetes-specific constructs distinct and avoids inappropriate meta-analysis.

The principal methodological limitation is the automation-assisted early title/abstract pathway. The *post hoc* random validation found no eligible record among 200 early exclusions, with complete agreement between the two reviewers, but only 200 of 3,321 early exclusions were sampled; the Wilson 95% upper confidence bound of 1.88% means residual false-negative risk cannot be excluded. Full text was available to the authors for only six of 40 included reports. For the remaining 34, some eligibility and descriptive variables relied on structured abstracts, public metadata or secondary evidence, and unavailable fields were not inferred. The full-text-only sensitivity analysis did not retain the favorable technology signals. Embase, CINAHL and APA PsycINFO were unavailable, and no formal design-specific risk-of-bias appraisal was performed. Small samples, cross-sectional designs, low response, overlapping cohorts, selected survivors and incomplete diabetes definitions further limit inference.

### 4.6 Research priorities

- Use explicit type 3c diagnostic criteria and preserve aetiology-specific reporting.
- Report total, completion-total and specific partial resections separately and distinguish pre-existing from new-onset diabetes.
- Agree a core outcome set spanning generic HRQoL, diabetes burden, hypoglycaemia, exocrine/nutritional symptoms and function.
- Evaluate measurement properties and clinically meaningful change for EQ-5D-5L, ADDQoL, PAID and DTSQ in type 3c diabetes, and evaluate SARC-F only as a candidate instrument requiring validation against objective sarcopenia measures.
- Integrate PROMs with CGM, pancreatic enzyme replacement, nutrition, muscle strength and body composition.
- Include adequately powered direct comparisons with type 1 and type 2 diabetes.

## 5 Conclusion

Patient-reported burden in type 3c diabetes is multidimensional and cannot be inferred from glycaemic outcomes alone. Overall health and physical function may be impaired after total pancreatectomy even when some PAID, ADDQoL or treatment-satisfaction findings resemble type 1 or other insulin-treated diabetes. Preliminary technology signals are hypothesis-generating because the favorable CFRD reports were small and available only as abstracts. EQ-5D-5L remains uncommon, ADDQoL is the most broadly used diabetes-specific quality-of-life measure, and SARC-F is absent and unvalidated in this population. Future studies should combine complementary generic, diabetes-specific and objective functional measures and report pancreatic aetiology, diabetes timing and resection extent explicitly.

## Data Availability

The original contributions presented in the study are included in the article/**Supplementary Material**. Further inquiries can be directed to the corresponding author.

## Glossary

ADDQoL: Audit of Diabetes-Dependent Quality of Life
AI: artificial intelligence
AID: automated insulin delivery
APA: American Psychological Association
CF: cystic fibrosis
CFAbd-Score: Cystic Fibrosis Abdominal Score
CFQ-R: Cystic Fibrosis Questionnaire–Revised
CFRD: cystic-fibrosis-related diabetes
CGM: continuous glucose monitoring
CI: confidence interval
CINAHL: Cumulative Index to Nursing and Allied Health Literature
DOI: digital object identifier
DSMQ: Diabetes Self-Management Questionnaire
DTSQ: Diabetes Treatment Satisfaction Questionnaire
EORTC: European Organisation for Research and Treatment of Cancer
EQ-5D-5L: EuroQol 5-Dimension 5-Level Questionnaire
GAD-7: 7-item Generalized Anxiety Disorder scale
HADS: Hospital Anxiety and Depression Scale
HbA1c: glycated haemoglobin A1c
HRQoL: health-related quality of life
ICTRP: International Clinical Trials Registry Platform
JBI: Joanna Briggs Institute
GLP-1 RA: glucagon-like peptide-1 receptor agonist
MEDLINE: Medical Literature Analysis and Retrieval System Online
MNA: Mini Nutritional Assessment
PAID: Problem Areas in Diabetes
PAID-20: 20-item Problem Areas in Diabetes scale
PANQOLI: Pancreatitis Quality of Life Instrument
PCC: Population–Concept–Context
PGIC: Patient Global Impression of Change
PHQ-8/PHQ-9: 8-/9-item Patient Health Questionnaire
PMID: PubMed identifier
PRISMA-ScR: Preferred Reporting Items for Systematic reviews and Meta-Analyses extension for Scoping Reviews
PROM: patient-reported outcome measure
QLQ-C30: EORTC Quality of Life Questionnaire Core 30
QLQ-PAN26: EORTC Quality of Life Questionnaire Pancreatic Cancer Module 26
SARC-F: Strength, Assistance with walking, Rise from a chair, Climb stairs and Falls
SF-12/SF-36: 12-/36-item Short Form Health Survey
SMBG: self-monitoring of blood glucose
SWiM: Synthesis Without Meta-analysis
TPIAT: total pancreatectomy with islet autotransplantation
WHO: World Health Organization
