# Supplementary material for: Patient-reported outcomes in adults with type 3c diabetes: a scoping review across aetiologies and pancreatic resection extent
Source: Front Endocrinol (Lausanne). 2026 Sep 14;17:1963887. doi: 10.3389/fendo.2026.1963887 (PMC13616728; doi:10.3389/fendo.2026.1963887)
Supplement: Supplementary file 1 [file Supplementaryfile1.docx]

Supplementary Material

Supplementary Table S1. Master evidence map of the 40 included reports

| **Report / design** | **Aetiology and operational type 3c basis** | **Resection and diabetes timing** | **Relevant sample / comparator** | **PROM(s) / follow-up** | **Evidence basis** | **Key finding** | **Main limitation / cohort overlap** |
| --- | --- | --- | --- | --- | --- | --- | --- |
| Grancini V. (2026) Prospective pre–post cohort | CFRD | Established insulin-treated CFRD; no resection | n=21; within-person baseline versus 3 months | DTSQ; ADDQoL; follow-up: 3 months | Structured abstract/public metadata; full text unavailable to authors | DTSQ 22→33 and ADDQoL −2.1→−0.9 after 3 months of AID (both p<0.001). | Small uncontrolled cohort; age range and PROM completeness require full-text confirmation. Full text was unavailable to the authors. |
| Martini R. (2026) Prospective single-arm intervention | CFRD | Established insulin-treated CFRD; no resection | n=19; baseline, 3 months of CGM and 3 months after discontinuation | PAID; follow-up: 3-month intervention plus 3 months after discontinuation | Structured abstract/public metadata; full text unavailable to authors | PAID 29→16 after 3 months of CGM (p=0.02), 19 three months after discontinuation. | Small uncontrolled cohort; missing-data handling and PAID scoring require full-text confirmation. Full text was unavailable to the authors. |
| Davidsen L. (2025) Randomized open-label crossover trial | Chronic pancreatitis | Diabetes secondary to established chronic pancreatitis; resection not required | 30 randomized; 29 completed; PROM n=25; CGM versus SMBG crossover | EORTC QLQ-C30; PGIC; Clarke; follow-up: 50-day CGM and SMBG periods separated by 20-day washout | Accessible full text | No significant EORTC QLQ-C30, PGIC or Clarke differences between CGM and SMBG. | Four participants did not provide PROM data; trial not powered for individual PROM domains. |
| Sunsoa H. (2024) Randomized mixed-methods pilot | CFRD | Established insulin-treated CFRD; no resection | 18 completed; structured education versus usual individual education | CFQ-R; PAID; follow-up: 12 weeks | Structured abstract/public metadata; full text unavailable to authors | CFQ-R physical, emotional, eating, role and respiratory domains improved after structured education; no between-group PAID difference. | Small pilot; group-specific numerical CFQ-R results require full-text confirmation. Full text was unavailable to the authors. |
| Johnston P.C. (2024) Clinical cohort with cross-sectional survey | Chronic pancreatitis | Diabetes explicitly secondary to chronic pancreatitis; 19% had prior pancreatic surgery | n=63; psychological PROM n=27; no external comparator | PANQOLI; GAD-7; PHQ-9; follow-up: PROM cross-sectional | Structured abstract/public metadata; full text unavailable to authors | PANQOLI mean 64.9/103; 16/27 exceeded both anxiety and depression thresholds. | Psychological PROM subset was small; PANQOLI scoring detail unavailable. Full text was unavailable to the authors. |
| Marchegiani G. (2022) Retrospective comparative cohort | Post-pancreatectomy | Converted total pancreatectomy versus high-risk pancreatoduodenectomy; complete endocrine insufficiency after total resection | Converted total pancreatectomy n=86 versus high-risk pancreatoduodenectomy n=101 | EORTC QLQ-C30/PAN26; diabetes-specific QoL; follow-up: 12 months | Structured abstract/public metadata; full text unavailable to authors | General, cancer and pancreas QoL were similar; diabetes-specific QoL was worse after C-TP. | Non-random surgical comparison; detailed instrument results unavailable to the authors. Full text was unavailable to the authors. |
| Shaw K. (2021) Cross-sectional analysis of long-term survivors | Post-pancreatectomy | Mixed partial resections; only postoperative/new-onset diabetes findings interpreted | Diabetes n=34; diabetes versus no diabetes and pre-existing versus new-onset diabetes | EORTC; ADDQoL; follow-up: Median 9.3 years after surgery | Structured abstract/public metadata; full text unavailable to authors | EORTC findings were similar by diabetes status; ADDQoL indicated that life would be better without diabetes; restricted interpretation. | Pre-existing and new-onset diabetes required separation; survivor selection. Full text was unavailable to the authors. |
| Latenstein A.E.J. (2021) Multicentre cross-sectional study | Post-pancreatectomy | New-onset diabetes more than 3 years after pancreatoduodenectomy or left pancreatectomy | New-onset diabetes n=22; population norms and insufficiency subgroups | PAID-20; EQ-5D-5L; EORTC (restricted); follow-up: Median 6.3 years | Structured abstract/public metadata; full text unavailable to authors | PAID-20 median 6.9/20; generic EQ-5D and EORTC outcomes were not separable for the diabetes subgroup. | Generic PROMs were not always separable for the diabetes subgroup. Full text was unavailable to the authors. |
| Stoop T.F. (2020) Cross-sectional post-total-pancreatectomy cohort | Post-pancreatectomy | Complete endocrine and exocrine insufficiency after total pancreatectomy | n=53; population norms and preoperative subgroup n=39 | EORTC QLQ-C30; PAID; DTSQ; follow-up: Median 21 months | Structured abstract/public metadata; full text unavailable to authors | EORTC global QoL 66.7 vs 76.4 in the general population; PAID burnout 13.2%; DTSQ median 28. | Cross-sectional survivor cohort; PAID burnout definition varied. Full text was unavailable to the authors. |
| You, L (2020) Retrospective comparative cohort | Post-pancreatectomy | Total versus tail-preserving subtotal resection; complete endocrine insufficiency after total resection | Total n=38 versus tail-preserving subtotal n=18 | EORTC QLQ-C30/PAN26; follow-up: To May 2019 or death | Accessible full text | Global health was similar; subtotal resection favoured functioning, symptoms, dietary restriction and future confidence. | Non-random surgical comparison; numerical EORTC domains incompletely accessible. |
| Kuo H.-J. (2019) Propensity-matched cross-sectional study | Post-pancreatectomy | Pancreatogenic diabetes after total pancreatectomy or pancreatoduodenectomy | Matched sample: total n=13 versus pancreatoduodenectomy n=26 | EORTC QLQ-C30; fatigue measures; follow-up: Cross-sectional; time from surgery not reported in abstract | Structured abstract/public metadata; full text unavailable to authors | Global QoL and fatigue intensity were similar; TP was associated with more fatigue interference, worse physical function and insomnia. | Small matched sample; time since surgery not reported in accessible source. Full text was unavailable to the authors. |
| Scholten L. (2019) National retrospective cohort with cross-sectional PROM | Post-pancreatectomy | Pancreatogenic diabetes after total pancreatectomy | PROM n=60; population norms and type 1 diabetes | EQ-5D-5L; PAID; DTSQ; ADDQoL; follow-up: Median 36 months | Structured abstract/public metadata; full text unavailable to authors | EQ-5D index 0.83 vs 0.87 in the general population; diabetes distress and satisfaction were similar to type 1 diabetes. | Retrospective survivor cohort; possible overlap with other Dutch surgical reports. Full text was unavailable to the authors. |
| Pulvirenti A. (2019) Two-centre retrospective cohort with cross-sectional PROM | Post-pancreatectomy | Pancreatogenic diabetes after total pancreatectomy | QoL n=94; population norms and within-cohort factors | SF-36; ADDQoL; follow-up: Median 63 months | Structured abstract/public metadata; full text unavailable to authors | SF-36 physical and mental component scores were lower than population norms. | Selected long-term survivors; numerical ADDQoL details unavailable. Full text was unavailable to the authors. |
| Kwong E. (2019) Cross-sectional comparative study | CFRD | Established CFRD; no resection | CFRD subgroups versus impaired and normal glucose tolerance; subgroup n not reported in abstract | CFQ-R; follow-up: Cross-sectional | Structured abstract/public metadata; full text unavailable to authors | CFQ-R treatment burden was worse with insulin-treated CFRD (p<0.001 after adjustment). | Small CFRD subgroups and residual confounding by CF severity. Full text was unavailable to the authors. |
| Wu W. (2016) Retrospective survivor cohort with cross-sectional survey | Post-pancreatectomy | Insulin- and enzyme-treated diabetes after total pancreatectomy | 36/100 eligible survivors responded; population norms, preoperative status and other insulin-dependent diabetes | SF-36; ADDQoL; follow-up: Median 5.9 years | Structured abstract/public metadata; full text unavailable to authors | SF-36 was impaired relative to population norms; ADDQoL was negative but mostly similar to other insulin-dependent diabetes. | 36% response among eligible survivors; substantial selection bias risk. Full text was unavailable to the authors. |
| Roberts K.J. (2014) Matched cross-sectional comparison | Post-pancreatectomy | Pancreatogenic diabetes after total pancreatectomy | Total-pancreatectomy responders n=28; matched type 1 diabetes controls | EORTC QLQ-C30/PAN26; PAID; follow-up: Long-term; exact interval not reported in abstract | Structured abstract/public metadata; full text unavailable to authors | General and pancreas-specific QoL were worse than in type 1 diabetes; only 1 of 20 PAID items was worse. | Small matched sample; time since surgery and one differing PAID domain require confirmation. Full text was unavailable to the authors. |
| Epelboym I. (2014) Retrospective cohort with cross-sectional PROM comparison | Post-pancreatectomy | Total resection; partial-resection results restricted to the postoperative-diabetes subgroup | Total n=17 versus pancreatoduodenectomy n=14 survey responders | EORTC QLQ-C30/PAN26; ADDQoL; follow-up: Cross-sectional after operations performed 1994–2011 | Structured abstract/public metadata; full text unavailable to authors | No significant EORTC or ADDQoL differences; travel and physical activity were the most affected domains. | Small groups; partial-resection ADDQoL findings may not be separable by diabetes status. Full text was unavailable to the authors. |
| Havermans T. (2009) Cross-sectional study | CFRD | Insulin-treated CFRD; no resection | CFRD n=10 versus CF without CFRD n=47 | CFQ-R; follow-up: Cross-sectional | Accessible full text | CFQ-R Weight score was 43.65 in CFRD vs 69.88 without CFRD (p=0.005). | Very small CFRD subgroup; residual confounding by CF severity. |
| Tierney S. (2008) Cross-sectional comparison with prospective hypoglycaemia diary | CFRD | Established CFRD; no resection | CFRD versus type 1 diabetes; group sizes not reported in accessible abstract | ADDQoL; hypoglycaemia experience; follow-up: Cross-sectional questionnaire plus prospective hypoglycaemia diary | Structured abstract/public metadata; full text unavailable to authors | Diabetes-related HRQoL was less negative and severe hypoglycaemia experience less frequent in CFRD than in type 1 diabetes. | Group sizes and questionnaire validation details unavailable to the authors. Full text was unavailable to the authors. |
| Billings B.J. (2005) Retrospective survivor cohort with cross-sectional survey | Post-pancreatectomy | Pancreatogenic diabetes after total pancreatectomy | 27/34 eligible survivors; population norms and other insulin-dependent diabetes | SF-36; ADDQoL; follow-up: Mean 7.5 years | Structured abstract/public metadata; full text unavailable to authors | SF-36 role-physical and general health scores were worse than population norms; ADDQoL was similar to other insulin-dependent diabetes. | Small survivor cohort; comparator definition and domain-level results incompletely accessible. Full text was unavailable to the authors. |
| Müller MW (2007) Prospective cohort | After total pancreatectomy; mixed indications | Diabetes/endocrine insufficiency after total pancreatectomy | NR in accessible source; comparator recorded where reported in the key finding | EORTC QLQ-C30; Follow-up NR in accessible source | Structured abstract/public metadata; full text unavailable to authors | Prospective cohort after total pancreatectomy; all participants required insulin and pancreatic enzyme replacement, and quality of life was measured with a named validated PROM. | Possible overlap with the Heidelberg cohort reported by Hartwig 2015. Full text was unavailable to the authors. |
| Hartwig W (2015) Long-term surgical cohort | After total pancreatectomy for neoplasms | Diabetes/endocrine insufficiency after total pancreatectomy | NR in accessible source; comparator recorded where reported in the key finding | EORTC QLQ-C30; EORTC QLQ-PAN26; Follow-up NR in accessible source | Bibliographic/secondary evidence; full text unavailable to authors | Large cohort after total pancreatectomy with long-term quality of life assessed using validated EORTC questionnaires. | Possible overlap with the Heidelberg cohort reported by Müller 2007. Full text was unavailable to the authors. |
| Oh MY (2021) Longitudinal surgical cohort | After total pancreatectomy | Diabetes/endocrine insufficiency after total pancreatectomy | NR in accessible source; comparator recorded where reported in the key finding | EORTC QLQ-C30; EORTC QLQ-PAN26; MNA; Follow-up NR in accessible source | Accessible full text | Direct assessment of long-term quality of life after total pancreatectomy using validated instruments; complete endocrine insufficiency was unambiguous in this group. | No obvious cohort overlap identified. |
| Stoop TF (2022) Propensity-score comparative cohort | Total versus partial pancreatectomy | Diabetes/endocrine insufficiency after total pancreatectomy | NR in accessible source; comparator recorded where reported in the key finding | EORTC QLQ-C30; EQ-5D-5L; Follow-up NR in accessible source | Structured abstract/public metadata; full text unavailable to authors | Validated PROMs and a separately reported total-pancreatectomy group allowed a direct comparison by resection extent. | Probable partial overlap with the Dutch Stoop 2020 cohort; treated as a related report. Only the separately interpretable subgroup or PROM was charted. Full text was unavailable to the authors. |
| Umman V (2023) Single-centre cohort | After total pancreatectomy | Diabetes/endocrine insufficiency after total pancreatectomy | NR in accessible source; comparator recorded where reported in the key finding | EORTC QLQ-C30; Follow-up NR in accessible source | Accessible full text | Adult single-centre cohort after total pancreatectomy with a validated quality-of-life questionnaire and metabolic covariates. | No obvious cohort overlap identified. |
| Vitale RJ (2026) Cross-sectional cohort | Cystic-fibrosis-related diabetes | Established CFRD; resection not applicable | NR in accessible source; comparator recorded where reported in the key finding | CF-Medication Beliefs Questionnaire; Daily Care Check-in; PHQ-8; GAD-7; BRIEF; Follow-up NR in accessible source | Structured abstract/public metadata; full text unavailable to authors | A separate CFRD subgroup (n=131) completed named patient-reported psychological and self-management instruments. | No obvious cohort overlap identified. Full text was unavailable to the authors. |
| Waage A (2025) Observational cohort | TPIAT; all participants remained insulin treated | Timing NR in accessible source | NR in accessible source; comparator recorded where reported in the key finding | EORTC QLQ-C30; Follow-up NR in accessible source | Structured abstract/public metadata; full text unavailable to authors | Small prospective adult TPIAT cohort; because all participants remained insulin treated, the postoperative diabetes group was separately identifiable. | No obvious cohort overlap identified. Full text was unavailable to the authors. |
| Nichols N (2024) Cross-sectional observational study | Cystic-fibrosis-related diabetes | Established CFRD; resection not applicable | NR in accessible source; comparator recorded where reported in the key finding | Validated tinnitus and balance questionnaires; Follow-up NR in accessible source | Structured abstract/public metadata; full text unavailable to authors | Adult cystic-fibrosis cohort with a separate CFRD group and directly reported symptoms using validated tinnitus and balance questionnaires; relevant as symptom PROMs rather than generic HRQoL. | No obvious cohort overlap identified. Full text was unavailable to the authors. |
| Ukegjini K (2024) Comparative surgical cohort | Total versus partial pancreatectomy; preoperative diabetes | Diabetes/endocrine insufficiency after total pancreatectomy | NR in accessible source; comparator recorded where reported in the key finding | EORTC QLQ-C30; Follow-up NR in accessible source | Structured abstract/public metadata; full text unavailable to authors | The total-pancreatectomy group had complete postoperative endocrine insufficiency and was separately reported. Partial-resection groups were not automatically classified as type 3c because diabetes predated surgery. | No obvious cohort overlap identified. Only the separately interpretable subgroup or PROM was charted. Full text was unavailable to the authors. |
| Caley LR (2023) Cross-sectional study | Cystic-fibrosis-related diabetes | Established CFRD; resection not applicable | NR in accessible source; comparator recorded where reported in the key finding | CFAbd-Score; Follow-up NR in accessible source | Structured abstract/public metadata; full text unavailable to authors | A validated cystic-fibrosis-specific gastrointestinal PROM was compared separately in adults with CFRD (n=27) and without CFRD. | No obvious cohort overlap identified. Full text was unavailable to the authors. |
| Kumar S (2023) Cross-sectional study | Cystic-fibrosis-related diabetes | Established CFRD; resection not applicable | NR in accessible source; comparator recorded where reported in the key finding | PAID; HADS; CFQ-R; Follow-up NR in accessible source | Structured abstract/public metadata; full text unavailable to authors | The adult CFRD population was explicit and named validated psychosocial and quality-of-life instruments were used. | No obvious cohort overlap identified. Full text was unavailable to the authors. |
| Dasgupta R (2022) Cross-sectional study | Fibrocalculous pancreatic diabetes | Established fibrocalculous pancreatic diabetes; resection not applicable | NR in accessible source; comparator recorded where reported in the key finding | Hypoglycemia Unawareness Index; Follow-up NR in accessible source | Accessible full text | Open full text confirmed use of the standardized eight-item Clarke questionnaire for hypoglycaemia awareness in adults with fibrocalculous pancreatic diabetes. | No obvious cohort overlap identified. |
| Balduzzi A (2020) Comparative cohort | New-onset diabetes after pancreatoduodenectomy | Partial-resection setting; only separately interpretable diabetes findings charted | NR in accessible source; comparator recorded where reported in the key finding | Diabetes Self-Management Questionnaire; PANQOLI; Graded Chronic Pain Scale; Follow-up NR in accessible source | Structured abstract/public metadata; full text unavailable to authors | The surgical group had a high prevalence of postoperative diabetes and DSMQ was reported comparatively; PANQOLI and pain findings were interpreted only where separable for the diabetes subgroup. | No obvious cohort overlap identified. Only the separately interpretable subgroup or PROM was charted. Full text was unavailable to the authors. |
| Currie S (2017) Cross-sectional study | Cystic-fibrosis-related diabetes | Established CFRD; resection not applicable | NR in accessible source; comparator recorded where reported in the key finding | Seven-Day Physical Activity Recall; Self-Care Inventory–Revised; Follow-up NR in accessible source | Structured abstract/public metadata; full text unavailable to authors | Included only adults with diagnosed CFRD and named patient-reported measures of physical activity and self-care; relevant to physical function and sarcopenia-related burden. | No obvious cohort overlap identified. Full text was unavailable to the authors. |
| Casadei R (2016) Prospective observational study | Total versus partial pancreatectomy | Diabetes/endocrine insufficiency after total pancreatectomy | NR in accessible source; comparator recorded where reported in the key finding | EQ-5D-5L; PAID; Follow-up NR in accessible source | Structured abstract/public metadata; full text unavailable to authors | Direct comparison of total and partial resection using validated generic and diabetes-specific PROMs; pancreatogenic diabetes after total resection was unambiguous. | Possible overlap with the Bologna cohort reported by Casadei 2010. Full text was unavailable to the authors. |
| Watanabe Y (2015) Long-term survivor cohort | After total pancreatectomy | Diabetes/endocrine insufficiency after total pancreatectomy | NR in accessible source; comparator recorded where reported in the key finding | SF-36v2; Follow-up NR in accessible source | Structured abstract/public metadata; full text unavailable to authors | Long-term survivors after total pancreatectomy completed SF-36v2; 96% independently managed pancreatogenic diabetes. | No obvious cohort overlap identified. Full text was unavailable to the authors. |
| Barbier L (2013) Surgical cohort | After total pancreatectomy | Diabetes/endocrine insufficiency after total pancreatectomy | NR in accessible source; comparator recorded where reported in the key finding | EORTC QLQ-C30; EORTC QLQ-PAN26; Follow-up NR in accessible source | Structured abstract/public metadata; full text unavailable to authors | Prospective assessment of long-term survivors after total pancreatectomy using validated EORTC instruments; diabetes status was unambiguous for the cohort. | No obvious cohort overlap identified. Full text was unavailable to the authors. |
| Dorlon M (2013) Observational TPIAT cohort | TPIAT; insulin-treated subgroup | Timing NR in accessible source | NR in accessible source; comparator recorded where reported in the key finding | SF-12; Follow-up NR in accessible source | Structured abstract/public metadata; full text unavailable to authors | The study directly related quality of life to new or increased insulin treatment; only findings for the clearly insulin-treated subgroup were considered. | Possible overlap with other reports from the same US TPIAT programme. Only the separately interpretable subgroup or PROM was charted. Full text was unavailable to the authors. |
| Casadei R (2010) Single-centre cohort | After total pancreatectomy | Diabetes/endocrine insufficiency after total pancreatectomy | NR in accessible source; comparator recorded where reported in the key finding | EORTC QLQ-C30 (confirmed in a secondary source); Follow-up NR in accessible source | Bibliographic/secondary evidence; full text unavailable to authors | The original total-pancreatectomy cohort reported quality of life using a named validated instrument; the review component of the publication was not extracted. | Possible overlap with the same-centre Casadei 2016 report. Full text was unavailable to the authors. |
| Behrman SW (2006) Retrospective surgical cohort | After total pancreatectomy for chronic pancreatitis | Diabetes/endocrine insufficiency after total pancreatectomy | NR in accessible source; comparator recorded where reported in the key finding | SF-36; Follow-up NR in accessible source | Structured abstract/public metadata; full text unavailable to authors | Seven adults after total pancreatectomy completed SF-36; postoperative pancreatogenic diabetes was unambiguous. | Small single-centre cohort; no obvious overlap identified. Full text was unavailable to the authors. |

Reports are mapped at publication level. Columns requested during peer review identify study design, operational type 3c basis, resection/diabetes timing, relevant sample and comparator, PROM and follow-up, evidence-access basis, key finding and major limitation or cohort-overlap concern. NR means not reported in the source accessible to the authors. Six reports were charted from accessible full text; 34 relied at least partly on structured abstracts, public metadata or secondary bibliographic evidence. No unavailable value was inferred. Several surgical reports may contain overlapping cohorts; participants were not summed across publications. Restricted inclusion means that only the separately interpretable diabetes subgroup or PROM was used.

Supplementary Table S2. Excluded reports and primary reasons

| **Report** | **Title** | **Primary exclusion reason** |
| --- | --- | --- |
| Ishola F.A. (2026) | Poor Glycemic Status Is Associated With Diabetes Distress After Total Pancreatectomy With Islet Autotransplantation | The cohort was eligible from age 16, and adult-only results were not reported separately. |
| Lane A. (2018) | Low prevalence of diabetes distress following total pancreatectomy with islet autotransplantation | The mean age was 34.3±15 years, indicating possible inclusion of minors; adult-only results were not reported separately. |
| Struyvenberg M.R. (2017) | Impact of Treatments on Diabetic Control and Gastrointestinal Symptoms after Total Pancreatectomy | No named or validated patient-reported outcome measure was identified; the report referred only to unspecified quality-of-life measures. |
| Benincá S.C. (2016) | Alcoholic chronic pancreatitis: A quality of life study | Diabetes was not clearly established as secondary/type 3c diabetes, type 2 diabetes could not be excluded, and no separable type 3c data were available. |
| Zakharchuk U.M. (2014) | [The parameters of estimation of the quality of life of patients with chronic pancreatitis and concomitant diabetes mellitus in outpatient practice] | Concomitant diabetes was not defined etiologically, and no separable data for confirmed type 3c diabetes were reported. |
| Lawton J (2026) | Participants' experiences of living with cystic fibrosis related diabetes and using a hybrid closed-loop system to support self-management: qualitative study | Qualitative interviews did not use a named standardized PROM and therefore did not meet the final instrument-based eligibility criterion. |
| Sherwood JS (2024) | Randomized trial of the insulin-only iLet bionic pancreas for the treatment of cystic fibrosis-related diabetes | Self-reported symptomatic hypoglycaemia was described, but the accessible report did not identify a named standardized PROM. |
| Coluzzi M (2023) | Improved Quality of Life Among Chronic Pancreatitis Patients Undergoing Total Pancreatectomy With Islet Autotransplantation—Single Center Experience With Large Cohort of Patients | Quality of life was reported for the whole TPIAT cohort, in which postoperative insulin dependence was not uniform; outcomes for a confirmed type 3c diabetes subgroup were not separable. |
| Takaki T (2023) | Quality of life after total pancreatectomy with islet autotransplantation for chronic pancreatitis in Japan | All five islet grafts remained functional at one year, and the accessible abstract did not establish that participants had diabetes or required insulin; type 3c outcomes were therefore not confirmed. |
| Phillips AE (2022) | Psychosocial outcomes 1-year post total pancreatectomy and autologous islet cell transplant | An adult subgroup was reported, but only 69.2% of adults were insulin dependent at one year and PROM findings were not stratified by diabetes status. |
| Ludwig S (2022) | Quality of life and metabolic outcomes after total pancreatectomy and simultaneous islet autotransplantation | Almost all participants retained at least partial endocrine function and PROM findings were not separable for a confirmed diabetes subgroup. |
| Marks BE (2021) | Perceptions of Diabetes Technology Use in Cystic Fibrosis-Related Diabetes Management | The study reported patient experiences using a bespoke 30-item online survey rather than a named standardized PROM. |
| Sherwood JS (2020) | Automated glycemic control with the bionic pancreas in cystic fibrosis-related diabetes: A pilot study | Treatment satisfaction and burden were reported, but the accessible information did not identify a standardized validated PROM. |
| Sutherland DER (2012) | Total pancreatectomy and islet autotransplantation for chronic pancreatitis | The cohort included children and adults with mixed endocrine outcomes; quality-of-life findings were not reported separately for adults with confirmed type 3c diabetes. |
| Drummond RS (2011) | Insulin therapy in patients with cystic fibrosis related diabetes mellitus: Benefit, timing of initiation and hypoglycaemia | The mixed population included participants aged 16–52 years, and results for adults aged at least 18 years were not separately extractable. |

Report not retrieved

Garcia A (2026), “Impact of Switching to Closed-loop Insulin Delivery System on Glycemic Control and Quality of Life in Adults Living With Cystic Fibrosis–related Diabetes.” Full text was not available and the public metadata did not identify a named standardized PROM; the report was not included in the synthesis.

Supplementary Appendix 1. Exact search strategies

MEDLINE via PubMed (PUBMED-V2)

*Run 2026-07-24*

( "type 3c diabetes"[tiab] OR "type 3c diabetes mellitus"[tiab] OR "pancreatogenic diabetes"[tiab] OR "pancreatic diabetes"[tiab] OR "diabetes of the exocrine pancreas"[tiab] OR "post-pancreatectomy diabetes"[tiab] OR ( ( diabet*[tiab] OR "Diabetes Mellitus"[Mesh] ) AND ( "Pancreatectomy"[Mesh] OR pancreatectom*[tiab] OR "pancreatic resection"[tiab] OR pancreatoduodenectom*[tiab] OR pancreaticoduodenectom*[tiab] OR "distal pancreatectomy"[tiab] OR "central pancreatectomy"[tiab] OR "total pancreatectomy"[tiab] OR "Pancreatitis, Chronic"[Mesh] OR "chronic pancreatitis"[tiab] OR "Pancreatitis, Acute Necrotizing"[Mesh] OR "acute pancreatitis"[tiab] OR "necrotizing pancreatitis"[tiab] OR "Pancreatic Neoplasms"[Mesh] OR "pancreatic cancer"[tiab] OR "pancreatic neoplasm"[tiab] OR "pancreatic neoplasms"[tiab] OR "Cystic Fibrosis"[Mesh] OR "cystic fibrosis"[tiab] OR "Hemochromatosis"[Mesh] OR haemochromatosis[tiab] OR hemochromatosis[tiab] OR fibrocalculous[tiab] OR "pancreatic trauma"[tiab] ) ) ) AND ( "Quality of Life"[Mesh] OR "Patient Reported Outcome Measures"[Mesh] OR "Surveys and Questionnaires"[Mesh] OR "Patient Satisfaction"[Mesh] OR "Fatigue"[Mesh] OR "quality of life"[tiab] OR "health-related quality of life"[tiab] OR hrqol[tiab] OR "patient-reported outcome"[tiab] OR "patient-reported outcomes"[tiab] OR "patient reported outcome"[tiab] OR "patient reported outcomes"[tiab] OR "patient-reported outcome measure"[tiab] OR "patient-reported outcome measures"[tiab] OR "patient reported outcome measure"[tiab] OR "patient reported outcome measures"[tiab] OR prom[tiab] OR proms[tiab] OR questionnaire*[tiab] OR self-report*[tiab] OR self report*[tiab] OR "health status"[tiab] OR "global health status"[tiab] OR "global impression of change"[tiab] OR pgic[tiab] OR "hypoglycemia awareness"[tiab] OR "hypoglycaemia awareness"[tiab] OR "Problem Areas in Diabetes"[tiab] OR "PAID score"[tiab] OR "PAID scale"[tiab] OR "PAID questionnaire"[tiab] OR dtsq[tiab] OR "EQ-5D"[tiab] OR "EQ-5D-5L"[tiab] OR addqol[tiab] OR "SARC-F"[tiab] OR "SF-36"[tiab] OR "SF-12"[tiab] OR eortc[tiab] OR "QLQ-C30"[tiab] OR "QLQ-PAN26"[tiab] OR pan26[tiab] OR "diabetes distress"[tiab] OR "fear of hypoglycemia"[tiab] OR "fear of hypoglycaemia"[tiab] OR "treatment satisfaction"[tiab] OR "treatment burden"[tiab] OR wellbeing[tiab] OR well-being[tiab] OR "physical functioning"[tiab] OR "social functioning"[tiab] OR "functional status"[tiab] OR fatigue[tiab] OR psychosocial[tiab] )

Validation note: 1,376 records. After two redundant zero-yield phrases were removed, PubMed displayed no phrase warnings. Sentinel check passed: Davidsen et al. was retrieved as PMID 40099620 (DOI 10.1111/dom.16356). No date or language limits were applied. Whitespace is compacted in this worksheet; Boolean logic and field tags are unchanged.

Scopus (SCOPUS-V2)

*Run 2026-07-23*

TITLE-ABS-KEY( "type 3c diabetes" OR "type 3c diabetes mellitus" OR "pancreatogenic diabetes" OR "pancreatic diabetes" OR "diabetes of the exocrine pancreas" OR "post-pancreatectomy diabetes" OR ( diabet* AND ( pancreatectom* OR "pancreatic resection" OR pancreatoduodenectom* OR pancreaticoduodenectom* OR "distal pancreatectomy" OR "central pancreatectomy" OR "total pancreatectomy" OR "chronic pancreatitis" OR "acute pancreatitis" OR "necrotizing pancreatitis" OR "pancreatic cancer" OR "pancreatic neoplasm" OR "pancreatic neoplasms" OR "cystic fibrosis" OR haemochromatosis OR hemochromatosis OR fibrocalculous OR "pancreatic trauma" ) ) ) AND TITLE-ABS-KEY( "quality of life" OR "health-related quality of life" OR hrqol OR "patient-reported outcome" OR "patient-reported outcomes" OR "patient reported outcome" OR "patient reported outcomes" OR "patient-reported outcome measure" OR "patient-reported outcome measures" OR "patient reported outcome measure" OR "patient reported outcome measures" OR prom OR proms OR questionnaire* OR "self-report" OR "self-reported" OR "self report" OR "self reported" OR "health status" OR "global health status" OR "global impression of change" OR pgic OR "hypoglycemia awareness" OR "hypoglycaemia awareness" OR "Problem Areas in Diabetes" OR "PAID score" OR "PAID scale" OR "PAID questionnaire" OR "PAID measure" OR dtsq OR "EQ-5D" OR "EQ-5D-5L" OR addqol OR "SARC-F" OR "SF-36" OR "SF-12" OR eortc OR "QLQ-C30" OR "QLQ-PAN26" OR pan26 OR "diabetes distress" OR "fear of hypoglycemia" OR "fear of hypoglycaemia" OR "treatment satisfaction" OR "treatment burden" OR wellbeing OR "well-being" OR "physical functioning" OR "social functioning" OR "functional status" OR fatigue OR psychosocial )

Validation note: 2,621 records. Sentinel check passed: Davidsen et al. (DOI 10.1111/dom.16356) was captured. The standalone acronym PAID was replaced with explicit PAID phrases to reduce irrelevant retrieval. Whitespace is compacted in this worksheet; Boolean logic and field tags are unchanged.

Web of Science Core Collection (WOS-V2)

*Run 2026-07-24*

TS=( "type 3c diabetes" OR "type 3c diabetes mellitus" OR "pancreatogenic diabetes" OR "pancreatic diabetes" OR "diabetes of the exocrine pancreas" OR "post-pancreatectomy diabetes" OR ( diabet* AND ( pancreatectom* OR "pancreatic resection" OR pancreatoduodenectom* OR pancreaticoduodenectom* OR "distal pancreatectomy" OR "central pancreatectomy" OR "total pancreatectomy" OR "chronic pancreatitis" OR "acute pancreatitis" OR "necrotizing pancreatitis" OR "pancreatic cancer" OR "pancreatic neoplasm" OR "pancreatic neoplasms" OR "cystic fibrosis" OR haemochromatosis OR hemochromatosis OR fibrocalculous OR "pancreatic trauma" ) ) ) AND TS=( "quality of life" OR "health-related quality of life" OR hrqol OR "patient-reported outcome" OR "patient-reported outcomes" OR "patient reported outcome" OR "patient reported outcomes" OR "patient-reported outcome measure" OR "patient-reported outcome measures" OR "patient reported outcome measure" OR "patient reported outcome measures" OR prom OR proms OR questionnaire* OR "self-report" OR "self-reported" OR "self report" OR "self reported" OR "health status" OR "global health status" OR "global impression of change" OR pgic OR "hypoglycemia awareness" OR "hypoglycaemia awareness" OR "Problem Areas in Diabetes" OR "PAID score" OR "PAID scale" OR "PAID questionnaire" OR dtsq OR "EQ-5D" OR "EQ-5D-5L" OR addqol OR "SARC-F" OR "SF-36" OR "SF-12" OR eortc OR "QLQ-C30" OR "QLQ-PAN26" OR pan26 OR "diabetes distress" OR "fear of hypoglycemia" OR "fear of hypoglycaemia" OR "treatment satisfaction" OR "treatment burden" OR wellbeing OR "well-being" OR "physical functioning" OR "social functioning" OR "functional status" OR fatigue OR psychosocial )

Validation note: 1,135 records. Web of Science Core Collection, Editions: All; no date, language or document-type limits. Sentinel check passed for Davidsen et al. (PMID 40099620). The export was downloaded in two batches; one overlapping boundary row was removed during the technical merge. Exact search history is retained in the uploaded search-history file. Whitespace is compacted in this worksheet; Boolean logic and field tags are unchanged.

ClinicalTrials.gov API v2 (CTGOV-V1)

*Run 2026-07-24*

( "type 3c diabetes" OR "pancreatogenic diabetes" OR "pancreatic diabetes" OR "diabetes of the exocrine pancreas" OR "post-pancreatectomy diabetes" OR ( diabetes AND ( pancreatectomy OR "pancreatic resection" OR "chronic pancreatitis" OR "acute pancreatitis" OR "pancreatic cancer" OR "pancreatic neoplasm" OR "cystic fibrosis" OR hemochromatosis OR haemochromatosis OR "pancreatic trauma" ) ) ) AND ( "quality of life" OR "health-related quality of life" OR "patient-reported outcome" OR questionnaire OR "EQ-5D" OR ADDQoL OR "SARC-F" OR "SF-36" OR "SF-12" OR EORTC OR "QLQ-C30" OR "QLQ-PAN26" OR "diabetes distress" OR "fear of hypoglycemia" OR "fear of hypoglycaemia" OR "treatment satisfaction" OR "treatment burden" OR wellbeing OR "well-being" OR fatigue OR psychosocial )

Validation note: 202 records. Official API v2, pageSize 1000, countTotal=true; no date, country, recruitment-status or study-type limits. All returned JSON records were retained. Davidsen NCT05550480 captured.

WHO ICTRP Search Portal (ICTRP-V1)

*Run 2026-07-24*

( "type 3c diabetes" OR "type 3c diabetes mellitus" OR "type3c diabetes" OR "type3c diabetes mellitus" OR T3cDM OR "pancreatogenic diabetes" OR "pancreatic diabetes" OR "diabetes of the exocrine pancreas" OR "post-pancreatectomy diabetes" OR "postpancreatectomy diabetes" OR "post-pancreatitis diabetes" OR "postpancreatitis diabetes" )

Validation note: The 12 exact terms shown in the OR block were executed separately because the public form was unstable with the complete long OR string; their union is logically equivalent. Results were combined and deduplicated by Main ID: 25 unique trial groups. Public detail records supplied primary and secondary outcomes. No date, country, recruitment-status or study-type limits. Davidsen NCT05550480 captured.

Supplementary Appendix 2. Selection-method transparency

The title/abstract pathway used rule-based eligibility signals and OpenAI ChatGPT/Codex to prioritize records. The initial candidate set contained 128 records. A subsequent audit reinstated 17 database records that the initial prioritization had not advanced and identified five reports outside the database exports. The final independently adjudicated report-level set contained 56 reports. The remaining 3,321 early exclusions were not initially dual-screened. On 27 August 2026, the authors completed a reproducible post hoc random validation sample of 200 records from this pool. Both reviewers independently applied the prespecified title/abstract criteria. They each classified 197 as exclude and the same three as unclear; exact three-category and binary agreement were 100% (Cohen's kappa = 1.00). Targeted adjudication excluded all three unclear records, leaving 0/200 eligible records (Wilson 95% confidence interval 0–1.88%). This audit estimates the potentially missed-eligible proportion in the sampled exclusion pool; it does not directly estimate screening sensitivity.

Supplementary Appendix 3. Post hoc validation of automation-assisted early exclusions

The validation sample and final adjudication were recorded in the authors' completed workbook. Sampling was deterministic and auditable; the exclusion-pool and sample hashes are retained in that workbook. Both reviewers confirmed that they screened the same 200 records independently before comparing decisions.

| **Item** | **Result** | **Interpretation** |
| --- | --- | --- |
| Early-exclusion pool | 3,321 records | Automation-assisted pathway |
| Validation sample | 200 records | Deterministic seed: Type3c_PROMs_posthoc_validation_2026-08-27_v1 |
| Independent decisions | Ana: 197 exclude, 0 retain, 3 unclear; Nika: identical | Decisions compared only after independent completion |
| Agreement | 100% exact and binary agreement; Cohen's kappa = 1.00 | Three-category and binary analyses |
| DB-01100 | Exclude | No standardized PROM in the 2021 record; quality-of-life outcomes were reported separately in 2023 |
| DB-01603 | Exclude | SF-36 used, but diabetes was not temporally or etiologically established as pancreatitis-related/type 3c |
| DB-03176 | Exclude | No accepted quality-of-life instrument and no separately reported diabetic/type 3c PROM subgroup |
| Final validation result | 0/200 eligible | Observed missed-eligible proportion 0%; Wilson 95% confidence interval 0–1.88% |

Supplementary Appendix 4. Abbreviation glossary

Abbreviations used in the manuscript, evidence tables and electronic-search documentation are listed alphabetically below.

| **Abbreviation** | **Meaning** | **Abbreviation** | **Meaning** |
| --- | --- | --- | --- |
| ADDQoL | Audit of Diabetes-Dependent Quality of Life | MNA | Mini Nutritional Assessment |
| AI | artificial intelligence | NCT | ClinicalTrials.gov study-identifier prefix |
| AID | automated insulin delivery | PAID | Problem Areas in Diabetes |
| APA | American Psychological Association | PAID-20 | 20-item Problem Areas in Diabetes scale |
| API | application programming interface | PAN26 | shorthand for EORTC QLQ-PAN26 |
| BRIEF | Behavior Rating Inventory of Executive Function | PANQOLI | Pancreatitis Quality of Life Instrument |
| C-TP | converted total pancreatectomy | PD | pancreatoduodenectomy |
| CF | cystic fibrosis | PGIC | Patient Global Impression of Change |
| CFAbd-Score | Cystic Fibrosis Abdominal Score | PHQ-8/PHQ-9 | 8-/9-item Patient Health Questionnaire |
| CFQ-R | Cystic Fibrosis Questionnaire–Revised | PMID | PubMed identifier |
| CFRD | cystic-fibrosis-related diabetes | PRISMA-ScR | Preferred Reporting Items for Systematic reviews and Meta-Analyses extension for Scoping Reviews |
| CGM | continuous glucose monitoring | PROM | patient-reported outcome measure |
| CINAHL | Cumulative Index to Nursing and Allied Health Literature | QLQ-C30 | EORTC Quality of Life Questionnaire Core 30 |
| CTGOV | ClinicalTrials.gov | QLQ-PAN26 | EORTC Quality of Life Questionnaire Pancreatic Cancer Module 26 |
| DOI | digital object identifier | QoL | quality of life |
| DSMQ | Diabetes Self-Management Questionnaire | SARC-F | Strength, Assistance with walking, Rise from a chair, Climb stairs and Falls |
| DTSQ | Diabetes Treatment Satisfaction Questionnaire | SF-12/SF-36 | 12-/36-item Short Form Health Survey |
| EORTC | European Organisation for Research and Treatment of Cancer | SF-36v2 | 36-item Short Form Health Survey, version 2 |
| EQ-5D-5L | EuroQol 5-Dimension 5-Level Questionnaire | SMBG | self-monitoring of blood glucose |
| GAD-7 | 7-item Generalized Anxiety Disorder scale | SWiM | Synthesis Without Meta-analysis |
| HADS | Hospital Anxiety and Depression Scale | T3cDM | type 3c diabetes mellitus |
| HbA1c | glycated haemoglobin A1c | tiab | PubMed title/abstract search field |
| HR-PD | high-risk pancreatoduodenectomy | TITLE-ABS-KEY | Scopus title, abstract and keyword search fields |
| HRQoL | health-related quality of life | TP | total pancreatectomy |
| ICTRP | International Clinical Trials Registry Platform | TPIAT | total pancreatectomy with islet autotransplantation |
| JBI | Joanna Briggs Institute | TS | Web of Science Topic search field |
| JSON | JavaScript Object Notation | V1/V2 | version 1/version 2 of the recorded search strategy |
| MEDLINE | Medical Literature Analysis and Retrieval System Online | WHO | World Health Organization |
| MeSH | Medical Subject Headings | WOS | Web of Science |
